# An Evasive Liver Mass in a Human Immunodeficiency Virus (HIV)-Positive Patient

**DOI:** 10.14309/crj.0000000000001481

**Published:** 2024-09-20

**Authors:** Reanay Berezovskiy, James M. Crawford, Arvind Rishi, Sohil Khurana, Joshua Kern, Stefani Morscher, Sanjaya K. Satapathy

**Affiliations:** 1 New York Institute of Technology College of Osteopathic Medicine, Old Westbury, NY; 2 Northwell Health, Hempstead, NY

**Keywords:** IgG4-related disease, immunosuppression, pseudotumor

## Abstract

IgG4-related disease (IgG4-RD) is an autoimmune syndrome that is characterized by elevated levels of serum IgG4 and infiltration of various tissue types by IgG4 immunoreactive plasma cells. The IgG4-RD can result in systemic disease and the formation of inflammatory mass lesions, frequently addressed as pseudotumors. While IgG4-RD can manifest in various organs, liver involvement is rare, and because it is an immune-mediated inflammatory process, it is uncommon in patients who are immunocompromised. Furthermore, despite IgG4-RD responding well to immunosuppressive treatment, cases of spontaneous remission are exceedingly rare in the literature. In this report, we present the unique case of a self-resolving IgG4-RD lesion of the liver in a HIV positive patient.

## INTRODUCTION

IgG4-related disease (IgG4-RD) has a complex pathophysiology that is not well understood. It is postulated that in IgG4-RD, 3 pathways converge: 1 involving activated B cells, another involving aberrant CD4^+^ cytotoxic T lymphocytes characterized by SLAMF7 expression, and a third involving M2 macrophages. These pathways collectively release cytokines that activate fibroblasts, stimulating the secretion of extracellular matrix proteins and driving tissue remodeling and fibrosis. Together these processes result in the formation of inflammatory tumor-like masses and organ enlargement.^1,2^ Typically, these masses respond well to immunosuppressive therapy, resolving within a few weeks to months of treatment initiation. The long-term progression of IgG4-RD remains relatively understudied due to prompt treatment upon diagnosis. However, the spontaneous resolution of our patient's mass highlights the need for further investigation into a potential self-limiting nature of IgG4-RD.

## CASE DESCRIPTION

**Figure 1.**
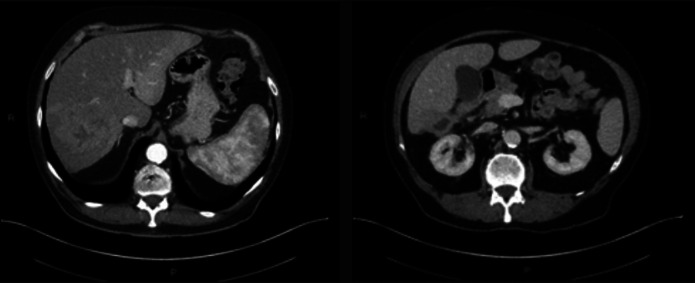
Arterial phase computed tomography demonstrated a large heterogenous mass in the right lobe of the liver measuring up to 11.2 cm (predominantly in segment 7 or 8). Areas of solid enhancement as well as a central necrosis are present. **IgG4-related pseudotumors usually present with variable enhancement patterns, most commonly exhibiting homogenous delayed enhancement.

**Figure 2.**
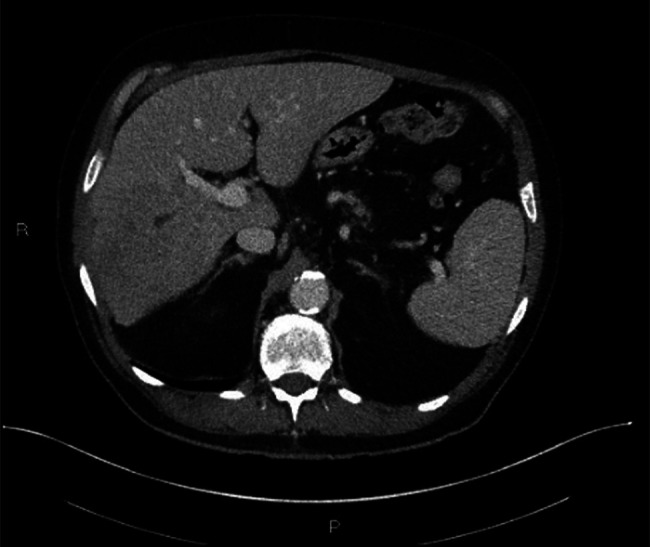
Venous phase computed tomography demonstrated right posterior portal vein thrombosis; raising suspicion for a tumor in vein.

A 76-year-old man with a history of HIV (viral load undetected; CD4 >200) on emtricitabine and dolutegravir therapy presented to the primary care provider following an unprovoked syncopal episode. Family history revealed cardiovascular-related deaths in both parents, with no history of liver disease. During the evaluation, the patient reported a decreased appetite, 10 lb weight loss, and a general feeling of unwellness. Initial laboratory tests revealed leukocytosis and elevated alkaline phosphatase levels. Physical examination was relevant for mild hepatomegaly. An arterial phase computed tomography scan revealed an 11.2 cm heterogeneous mass in the right lobe of the liver (Figure 1). Additionally, venous phase computed tomography revealed thrombosis in the right posterior portal vein, raising suspicion of a tumor within the vein (Figure 2). The location and size of the mass raised concerns for potential malignancy with the differential diagnosis including hepatocellular carcinoma, cholangiocarcinoma, abscess, metastatic disease, lymphoma, and hemangioma. The patient was initiated on a 2-week course of metronidazole as an empirical treatment for a potential infection manifesting as a liver abscess and was referred to a hepatology clinic for further evaluation.

Laboratory tests revealed leukocytosis (11.44 × 10^3^/µL) with neutrophilia (9.27 × 10^3^/μL; 81.0%), macrocytic anemia (red blood cell count = 3.39 × 10^6^/μL, hemoglobin = 10.2 g/dL, hematocrit = 34.5%, mean corpuscular volume = 101.8 fL), and elevated ferritin (1,403 μg/L). Chemistry was notable for elevated serum alkaline phosphatase (227 IU/L), gamma-glutamyl transpeptidase (129 IU/L), and normal transaminases. Tumor markers, viral, and autoimmune hepatitis serologies were within normal range aside from elevated alpha-1-antitrypsin (>300 mg/dL).

**Figure 3.**
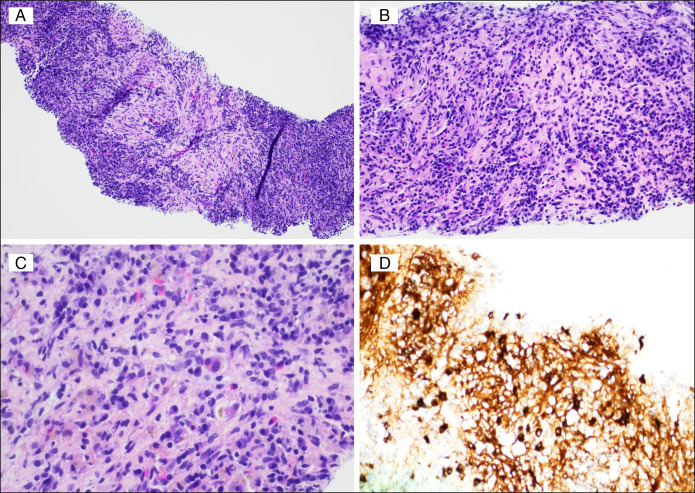
Densely inflamed liver biopsy tissue with storiform pattern of fibrosis (A 100× magnification H&E). Numerous plasma cells in a background of completely replaced hepatic parenchyma by the fibrosis (B 200× magnification H&E and (C) 400× magnification H&E). IgG4 immunohistochemistry highlight plasma cells within fibrotic stroma (400× magnification).

The patient underwent percutaneous needle biopsy followed by a fine needle biopsy 2 weeks later. The first biopsy was percutaneous needle biopsy, which showed small fragments of granulation tissue and histiocytic aggregates. No acid-fast bacilli or fungal microorganisms were identified on the first biopsy. This was followed up in 2 weeks by an endoscopic ultrasound-guided fine-needle aspiration biopsy performed through the duodenum. The second biopsy showed densely fibrous liver tissue with rare native reactive bile ducts. The fibrous areas showed storiform pattern of fibrosis (Figure 3A), numerous plasma cells (Figure 3B and 3C), which were polyclonal on Kappa and Lambda in situ hybridization. Immunoglobulin G4 (IgG4) immunohistochemistry showed about 25 IgG4 plasma cells per high-power field (Figure 2D), representing >40% of plasma cells identified by IgG immunohistochemistry (not shown). This high IgG4:IgG ratio of plasma cells was supportive of the criteria for a diagnosis of IgG4-RD and correlated with the persistently elevated serum IgG4. Acid-fast bacilli stain for mycobacteria and immunohistochemistry for human herpesvirus-8 and *Treponema pallidum* were negative. While a percutaneous image-guided core biopsy was inconclusive, an endoscopic ultrasound-guided fine-needle aspiration biopsy supported the diagnosis of IgG4-RD. This discrepancy highlights the potential for sampling error or heterogeneity within the lesion. It emphasizes the need for careful evaluation and biopsy of any unusual liver lesion, especially in immunocompromised individuals, in whom atypical presentations may occur.

**Figure 4.**
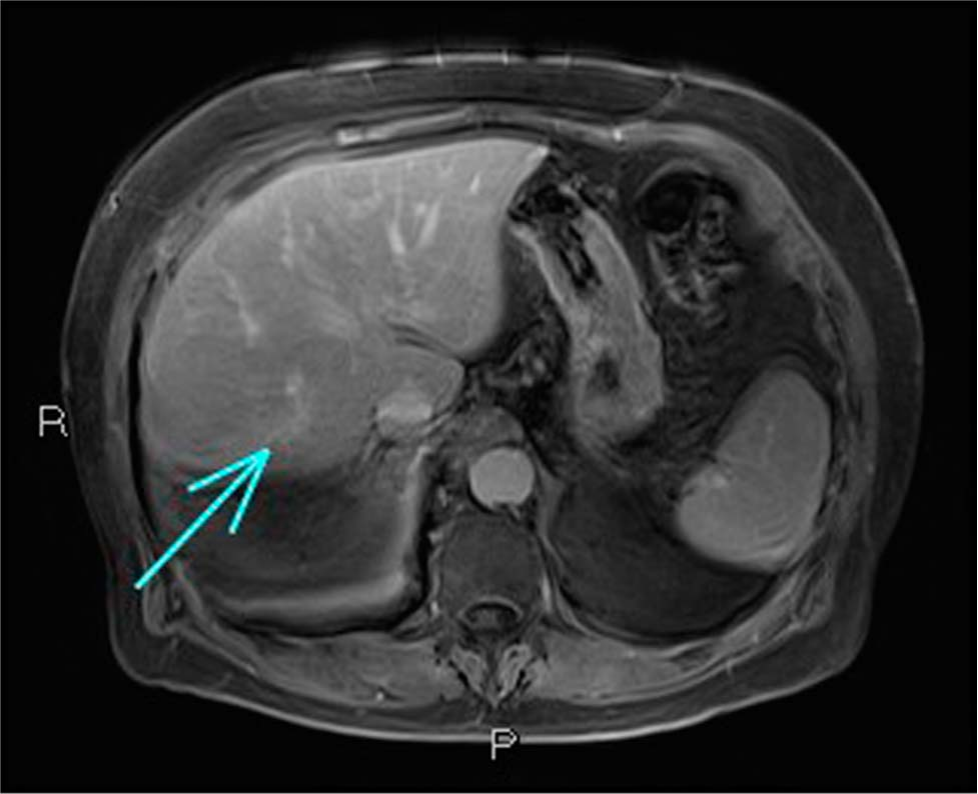
Magnetic resonance imaging at 18-month mark; enhancing scar at the site of previous lesion depicted by the arrow.

Follow-up imaging showed progressive regression of the liver mass and atrophy of the right hepatic lobe, suggesting a resolving process. At 18 months, the mass was resolved on imaging (Figure 4). Throughout the follow-up period, the patient remained asymptomatic and denied any abdominal pain, fever, or other concerning symptoms, indicating a stable clinical course. The elevated IgG4 levels persisted, warranting ongoing monitoring.

## DISCUSSION

IgG4-RD is characterized by its multiorgan involvement and tumor-like morphology. Predominantly, IgG4-RD affects the pancreas, bile ducts, salivary glands, lacrimal glands, kidneys, retroperitoneum, orbita, lymph nodes, and lungs.^3,4^ Studies have shown that the pancreas is most frequently involved in systemic IgG4 disease, with the biliary tract being the most common site of extrapancreatic involvement.^3–5^ Notable clinical presentations include hepatobiliary strictures caused by IgG4-related sclerosing cholangitis and IgG4-related pancreatitis. Although cases of IgG4-RD in the liver are rare, hepatic pseudotumor formation is recognized as a common presentation of this condition.^6^ It remains unclear whether the incidence of IgG4-RD in immunocompromised individuals differs from that of the general population. However, altered immune surveillance and response mechanisms in these patients can affect the disease course.

To diagnose IgG4-RD, histopathology and laboratory values must be taken into consideration. The Boston Consensus Criteria for IgG4-RD include key histopathological features such as dense lymphoplasmacytic infiltrate, storiform fibrosis, and obliterative venulitis. In patients with positive biopsy findings, plasma IgG4+/IgG ratios >40% and the number of IgG4 positive plasma cells greater than 10 per high powered field are definitive for IgG4-RD.^4^ It is important to note that elevated levels of IgG4+ in plasma can occur in various conditions, including pancreatic cancer, lymphoma, and gastrointestinal cancers. In addition, patients with IgG4-RD may have an increased risk of developing subsequent malignancies. Therefore, the measurement of serum IgG4 levels should be reserved for cases where suspicion for IgG4-RD is high and in which malignancy has been ruled out.^7,8^ In our patient, the confluence of findings, including chronically elevated IgG4 serum levels with an IgG4+/IgG ratio >40%, the presence of IgG4+ cells on liver biopsy, and the absence of malignant cells on biopsy led to a conclusive diagnosis of IgG4-RD of the liver.

Systemic glucocorticoids are the first-line treatment for the inflammatory component of IgG4-RD. However, monotherapy with glucocorticoids can result in relapse. Combining steroids, immunomodulators, and B-cell depletion agents such as rituximab can prevent relapse, with rituximab maintenance every 6 months showing the lowest relapse rate (∼10%). Recent studies have shown that T-follicular helper (Tfh2) cell levels were higher in patients with IgG4-RD and that Tfh2 counts correlate with the number of affected organs and disease activity. Unlike IgG4 serum concentrations, Tfh2 levels do not respond to glucocorticoid therapy and may contribute to disease relapse.^9^ Therefore, Tfh2 cells can be a potential target for future drug therapies.

In our patient's case, by the time they visited the hepatology clinic and underwent repeat imaging, the size of the mass had decreased. Given this spontaneous improvement and the undesirable side effects of steroids, the decision was made to defer treatment and monitor the patient. The spontaneous resolution of our patient's mass highlights the need for a nuanced approach to treatment and consideration of individual factors. Notably, there have been other reported case of spontaneous remission of an IgG4-related hepatic pseudotumor in patients with HIV,^10^ further suggesting the possibility for a self-limiting nature of IgG4-RD. The factors contributing to the spontaneous remission in these patients and the role that their immunocompromised status played in their remission warrants further investigation, as they could potentially reveal novel insights into the mechanisms underlying IgG4-RD. In particular, the immunomodulated state of a patient undergoing treatment for HIV raises the question both of how IgG4-RD could arise in the first place, and why it might then resolve spontaneously. Investigation of the effect of the antiviral therapies on plasma cell biology might be of interest, noting that circulating IgG4 levels play a role in maintaining viral control.^11^ Achieving the right balance between treating disease and avoiding unnecessary medical interventions is paramount, as the risks associated with certain treatments can be significant. These clinical scenarios emphasize a possibility for shifts from immediate intervention to comprehensive observation of IgG4-RD, with the understanding that treatment may potentially be deferred until it becomes necessary.

## DISCLOSURES

Author contributions: R. Berezovskiy wrote the manuscript; JM Crawford, S. Khurana, SK Satapathy: wrote and edited the manuscript; A. Rishi, J. Kern: edited the manuscript and provided images for figures; S. Morscher: Gathered information and edited the manuscript. SK Satapathy is guarantor of the article.

Financial disclosure: None to report.

Previous presentation: This study was presented at the American Association for the Study of Liver Diseases Case Conference; February 17, 2023; virtual.

Informed consent was obtained for this case report.
